# Root Cause? Yes of course … but then what?

**DOI:** 10.26633/RPSP.2019.51

**Published:** 2019-06-07

**Authors:** José Joaquín Mira Solves, Irene Carrillo, Mercedes Guilabert, José L Valencia-Martín, Jesús María Aranaz Andrés, Jimmy Martin

**Affiliations:** 1 Universidad Miguel Hernandez de Elche Universidad Miguel Hernandez de Elche Elche Spain Universidad Miguel Hernandez de Elche, Elche, Spain.; 2 Hospital Universitario Ramón y Cajal Hospital Universitario Ramón y Cajal Madrid Spain Hospital Universitario Ramón y Cajal, Madrid, Spain; 3 Foundation for the Promotion of Health and Biomedical Research Foundation for the Promotion of Health and Biomedical Research Sant Joan d’Alacant Spain Foundation for the Promotion of Health and Biomedical Research, Sant Joan d’Alacant, Spain

Dear Editor:

Analyzing the causes of unsafe care can reduce the number of ‘near misses’ (incidents that may cause harm to patients) and adverse events (that actually produce harm). This is an important mandate for health care organizations committed to providing a safe environment for patients. Although guaranteeing absolute safety in all interventions is not always possible, hospitals and other health care institutions implement safety practices and surveillance methods to understand how these unsafe incidents occur. In many cases, these incidents were not generated by a single cause; and remote causes are as significant as more proximate ones.

Root Cause Analysis (RCA) is a technique used globally across diverse disciplines to understand the causes of avoidable safety incidents (1). RCAs systematically identify the causes of problems in clinical settings but can also be applied to preventive medicine and public health contexts. RCAs are closely-related to theories of public health causality. For example, there have been cases of overdose of tuberculosis vaccine because the multipurpose package has been confused with the PPD (purified protein derivative) skin test, which is used as a diagnostic test for tuberculosis. The goal is to identify the root causes (both remote and recent) of safety incidents and the actions necessary to eliminate them. In sum, RCAs determine the error that happened, why it happened, and how to avoid a recurrence. However, various studies have encouraged us to reflect on the usefulness of RCAs by highlighting their limitations and suggesting areas for improvement (2-5).

Focusing on more proximate errors and excluding latent causes from the analysis only partially helps to avoid further incidents (2). In the case of adverse events, the RCA must always correctly distinguish between the historical and ongoing factors that caused the event. After identifying the source of the problem, proposing effective solutions becomes the next priority.

When human error is the main cause of safety incidents, there is a greater likelihood of resistance to change. Front-line professionals who care for patients, are often reluctant to participate in RCAs, not only because this technique is time-consuming, but because of uncertainty about possible repercussions both for participants and those implementing recommendations (2). The balance between independence for executing the analysis and transparency is not easily achieved.

To strengthen the relevance and effectiveness of RCAs, some methodological innovations have been proposed. These include proposals based on simulations for strengthening the usefulness of the root cause analysis such as the London Protocol (which is an established methodological assessment designed to identify the series of events that caused an incident to occur), or analyzing critical incidents that reduce the time required by professionals to invest in performing an RCA (3).

Results from root cause analyses have not always been sufficiently useful in preventing the recurrence of adverse incidents or ‘near misses’ (4). We argue that there ought to be differentiation between the two main processes of root cause analysis: (i) the identification of the problem and the analysis of its causes; and (ii) the implementation of actions to prevent the harm from being repeated. While the first step is successfully done in most RCAs, the second usually fails because it is tasked to another group of professionals within the organization who did not participate in the RCA. To effectively implement RCAs in daily practice, the interdependence of the two steps should be considered. In this way, during the second phase where preventive action is required, management professionals with greater decision-making capacity would be involved. This guarantees greater implementation of the measures proposed by the RCA.

Furthermore, those professionals most directly involved in incidents should participate to some degree, in the RCA (which could result in them feeling more involved in the solution) (5). Patients or their family members could also provide information for the analysis and be informed about its results—much like the open discussion policy following a severe adverse event.

These are strategies that have not been widely implemented as part of RCAs and can be the focus of future research. RCAs have solid historical and theoretical foundations with the potential for making meaningful impact to efforts to enhance patient safety. However, they can only be effective if they are supported by changes to the regulatory and safety culture of health care organizations.

## Disclaimer.

Authors hold sole responsibility for the views expressed in the manuscript, which may not necessarily reflect the opinion or policy of the RPSP/PAJPH and/or PAHO.

**José Joaquín Mira Solves**

**Irene Carrillo**

**Mercedes Guilabert** mguilabert@umh.es

Universidad Miguel Hernandez de Elche, Elche, Spain.

**José L. Valencia-Martín**

**Jesús María Aranaz Andrés**

Hospital Universitario Ramón y Cajal, Madrid, Spain

**Jimmy Martin**

Foundation for the Promotion of Health and Biomedical Research, Sant Joan d’Alacant, Spain
